# Enhanced Ammonia Decomposition by Tuning the Support Properties of Ni/Gd_x_Ce_1-x_O_2-δ_ at 600 °C

**DOI:** 10.3390/molecules28062750

**Published:** 2023-03-18

**Authors:** Haihua He, Chonglai Chen, Chaoqun Bian, Junhua Ren, Jiajia Liu, Wei Huang

**Affiliations:** College of Pharmacy, Jinhua Polytechnic, Jinhua 321007, China

**Keywords:** Ni catalyst, Ce_0.8_Gd_0.2_O_2-δ_ support, ammonia decomposition, hydrogen production

## Abstract

Ammonia decomposition is a promising method to produce high-purity hydrogen. However, this process typically requires precious metals (such as Ru, Pt, etc.) as catalysts to ensure high efficiency at relatively low temperatures. In this study, we propose using several Ni/Gd_x_Ce_1-x_O_2-δ_ catalysts to improve ammonia decomposition performance by adjusting the support properties. We also investigate the underlying mechanism for this enhanced performance. Our results show that Ni/Ce_0.8_Gd_0.2_O_2-δ_ at 600 °C can achieve nearly complete ammonia decomposition, resulting in a hydrogen production rate of 2008.9 mmol.g^−1^.h^−1^ with minimal decrease over 150 h. Density functional theory calculations reveal that the recombinative desorption of nitrogen is the rate-limiting step of ammonia decomposition over Ni. Our characterizations indicate that Ni/Ce_0.8_Gd_0.2_O_2-δ_ exhibits a high concentration of oxygen vacancies, highly dispersed Ni on the surface, and abundant strong basic sites. These properties significantly enhance the associative desorption of N and strengthen the metal support interactions, resulting in high catalytic activity and stability. We anticipate that the mechanism could be applied to designing additional catalysts with high ammonia decomposition performance at relatively low temperatures.

## 1. Introduction

Hydrogen is considered an important carrier of energy for the future due to its clean and renewable characteristics [1,2]. However, the low density and high flammability of hydrogen present significant challenges for its storage and transportation, severely hindering the scale-up of hydrogen fuel use [3,4]. To address the above-mentioned issues, researchers have proposed the use of liquid fuels with high energy density, such as methanol [5] and ammonia [6], as carriers for hydrogen [7]. Among all the candidates, ammonia represents a highly promising medium to store and produce hydrogen due to ammonia’s impressive energy density (3000 Wh/kg), ease of liquefaction (~0.8 MPa, 20 °C), low cost, safe storage and transport attributes, and well-established infrastructure [8,9,10]. Consequently, ammonia has gained increasing attention worldwide as a medium for hydrogen storage. An efficient ammonia decomposition reaction is a key factor in hydrogen storage. However, currently the ammonia decomposition reaction requires the use of noble metals (Ru, Pt, etc. [11]) as catalysts to ensure efficient reaction kinetics and high ammonia conversion at relatively low temperatures. The scalable utilization of ammonia as an intermediary to store hydrogen has been severely hindered by the high cost of these metals. Thus, it is crucially important to develop non-precious catalysts that can efficiently decompose ammonia at low temperatures.

Currently, the attention of numerous researchers has been directed toward transition-metal-based catalysts, such as Ni [12], Fe [13], and Co [14], for ammonia decomposition. Within these transition metals, Ni is the most active and shows high potential for practical application [15]. Nowadays, Ni has been widely recognized as an alternative to Ru-based catalysts, and it has been preliminary investigated [16]. However, Ni is not active enough, and its performance requires further improvement at medium or low temperatures. For example, in a recent study by Martinez and colleagues [17], Ru was incorporated into Ni-based catalysts to facilitate ammonia decomposition, resulting in simultaneously improved activity and stability. Meanwhile, Thomson and colleagues [18] investigated the effect of platinum (Pt) as an additive for Ni. Their findings suggest that the bimetallic PtNi catalyst can significantly alleviate the hydrogen poisoning effect, resulting in a much-improved performance. However, the use of precious metals as dopants would increase the cost and inevitably hinder the application of these catalysts. To overcome this problem, Xiao’s group [19] synthesized several bimetallic MNi/Al_2_O_3_ catalysts, using Co, Ni, and Fe as dopants to improve the catalytic activity and delved into the synergy between the dopants and Ni to disclose the mechanism for the enhanced performance. The results show that bimetallic CoNi/Al_2_O_3_ exhibits almost full ammonia decomposition at 700 °C with the stable trend in the temperature range of 650–750 °C. We can conclude from these results that the doping strategy can effectively enhance the performance of Ni. However, the catalytic performance is still unsatisfactory at temperatures <700 °C.

In addition to the doping strategy, the literature also suggests that the activity of Ni is contingent on the support, with the support properties exerting a significant influence on its ammonia decomposition performance [20,21]. Recent efforts have focused on depositing Ni on different supports [22,23]. Xiao’s group proposed the Ni/MgAl_2_O_4_ catalysts with different support morphologies and studied the effect of the support morphologies on performance [24]. The findings indicate that Ni/MgAl_2_O_4_-LDH exhibits superior ammonia decomposition performance due to its high surface area and well-dispersed Ni. Additionally, the porous alumina matrix’s structural features were observed by Wang et al. [25] to promote the production of finely dispersed and small-sized Ni particles, thereby resulting in improved catalytic performance. These studies imply that tuning the support properties can effectively facilitate the promotion of Ni’s catalytic activity. However, the synthesis of these supports requires sophisticated methods, unsuitable for the scale-up of catalysts’ synthesis. In addition, the origin of the enhanced performance by support modification is still unclear. 

In this study, we have synthesized several Ni/Gd_x_Ce_1-x_O_2-δ_ as catalysts for ammonia decomposition by tuning the properties of Gd_x_Ce_1-x_O_2-δ_ support. The results show both high ammonia conversion and stability for Ni/Gd_0.2_Ce_0.8_O_2-δ_ at 600 °C. The improved performance was studied through a combination of DFT calculation and different physicochemical characterizations, revealing the fundamental mechanism behind it.

## 2. Results

### 2.1. Characterizations

**Figure 1:**
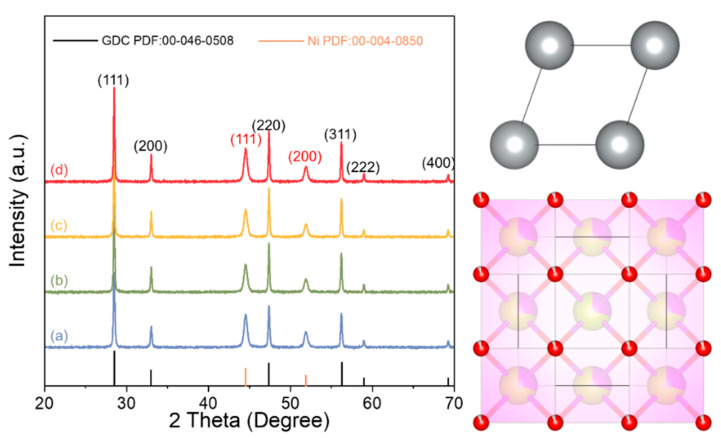
XRD patterns of the reduced Ni/Gd_x_Ce_1-x_O_2-δ_ catalysts. (a) Ni/Gd_0.1_Ce_0.9_O_2-δ_, (b) Ni/Gd_0.2_Ce_0.8_O_2-δ_, (c) Ni/Gd_0.3_Ce_0.7_O_2-δ_, and (d) Ni/Gd_0.4_Ce_0.6_O_2-δ_.

The X-ray diffraction (XRD) patterns in Figure 1 show that all catalysts exhibit a similar cubic fluorite structure of GDC (PDF: 00-046-0508) and Ni (PDF: 00-004-0850). The absence of gadolinium planes is attributed to its proper doping within the ceria lattice. Thus, it can be deduced that the Gd_x_Ce_1-x_O_2-δ_ supports and the active compound are in a crystalline phase. Importantly, based on the XRD patterns, there is no evidence of new compound formation or chemical reaction occurring between the support and the active compound. Scherer’s formula (Equation (1)) was used to evaluate crystallite sizes of the supports based on the (111) peak of Gd_x_Ce_1-x_O_2-δ_:

(1) $$D=\frac{0.89\lambda }{\beta \cos\theta }$$

where λ, β, θ, and D represent the X-ray wavelength, the angle between the sample and the X-ray beam, the full width at half maximum position, and the crystallite size, respectively. The derived crystallite size and lattice constant values of the support are summarized in Table 1. Specifically, we have found that the lattice constants of Gd_0.1_Ce_0.9_O_2-δ_, Gd_0.2_Ce_0.8_O_2-δ_, Gd_0.3_Ce_0.7_O_2-δ_, Gd_0.4_Ce_0.6_O_2-δ_ are 5.15 Å, 5.17 Å, 5.20 Å, and 5.23 Å. We can attribute this discrepancy to the larger ionic radii of Gd^3+^ (1.19Å) compared with Ce^3+^ (1.01Å), as previously reported in the literature [26].

**Figure 2:**
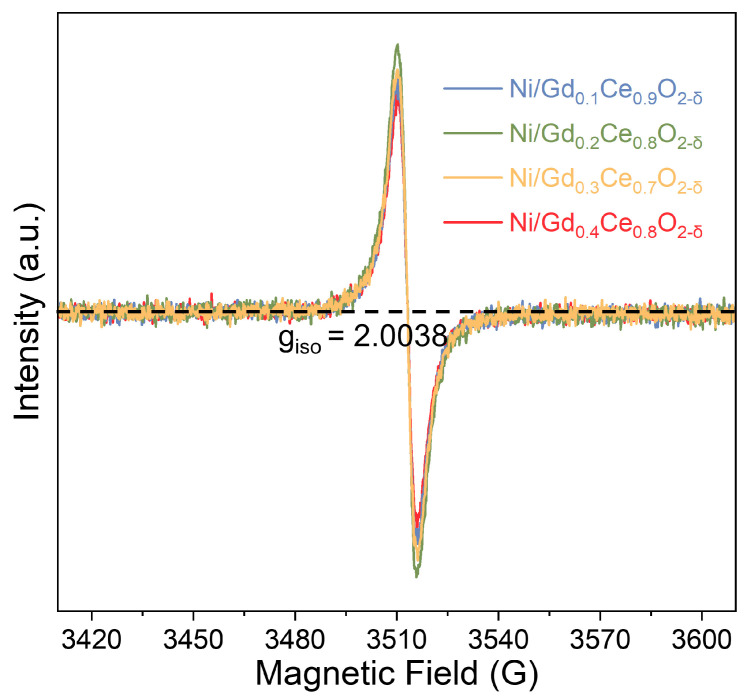
EPR profiles of the reduced Ni/Gd_x_Ce_1-x_O_2-δ_ support.

The EPR profiles displayed in Figure 2 reveal the oxygen vacancy concentration in the bulk of the reduced catalysts. The EPR signal centered at approximately 2.003 is present in all catalysts, indicating the existence of oxygen vacancies. The catalysts’ intensity follows this ranking: Gd_0.2_Ce_0.8_O_2-δ_ > Gd_0.3_Ce_0.7_O_2-δ_ > Gd_0.1_Ce_0.9_O_2-δ_ > Gd_0.4_Ce_0.6_O_2-δ_. This indicates that Gd_0.2_Ce_0.8_O_2-δ_ shows the most oxygen vacancies among all the samples. Therefore, we can conclude that adjusting the molar ratio of Gd and Ce can increase the oxygen vacancy concentration, with Gd_0.2_Ce_0.8_O_2-δ_ showing the highest concentration.

Figure S1 illustrates the SEM images of the Ni/Gd_x_Ce_1-x_O_2-δ_ with different Gd: Ce molar ratios. The results show the catalysts featured in the gathered spherical structures. In addition, the morphologies of the catalysts were found to be similar across all samples. This suggests that varying the molar ratio of Gd:Ce did not significantly impact the surface morphologies. Therefore, the difference in the catalytic activity was not influenced by the surface morphologies of the catalysts. 

Additional textural properties in terms of surface area, actual Ni loading, Ni dispersion, and Ni particle size are shown in Table 2. It can be seen that all catalysts show a similar surface area at around 120 m^2^/g. The actual Ni loading of all catalysts was estimated to be near 9.8%, which is only slightly lower than the nominal Ni amount. The Ni particle size of the catalysts was also not significantly influenced by the Gd: Ce molar ratio. However, comparing the Ni dispersion of the catalysts on the surface, we can find that Ni/Gd_0.2_Ce_0.8_O_2-δ_ displays the highest Ni dispersion of 20.7%. In addition, the amount of Ni dispersion is positively related to that of the oxygen vacancy concentration. This observation is consistent with recent findings by Liu et al. that the oxygen vacancy on the surface of catalysts can promote the dispersion of active sites on the surface [27].

**Figure 3:**
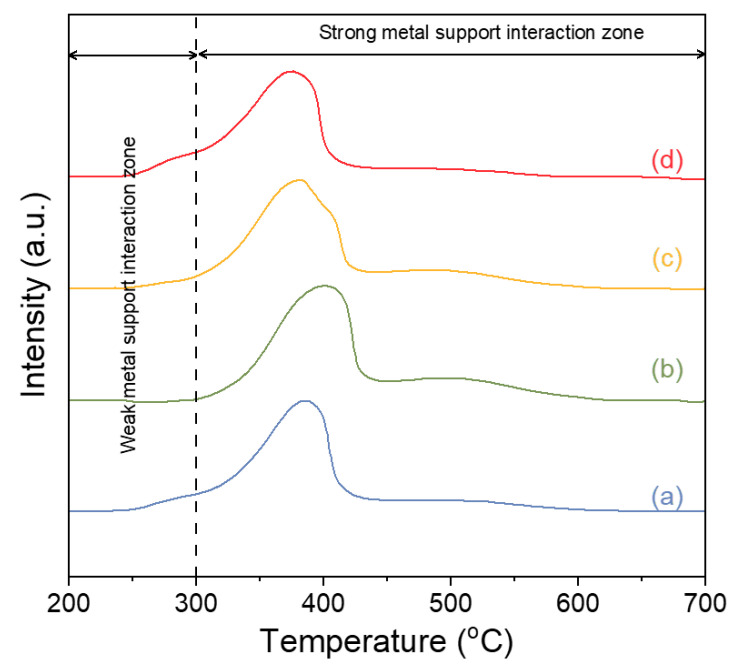
H_2_-TPR of the as-prepared NiO/Gd_x_Ce_1-x_O_2-δ_ catalysts. (a) NiO/Gd_0.1_Ce_0.9_O_2-δ_, (b) NiO/Gd_0.2_Ce_0.8_O_2-δ_, (c) NiO/Gd_0.3_Ce_0.7_O_2-δ_, and (d) NiO/Gd_0.4_Ce_0.6_O_2-δ_.

H_2_-TPR profiles of the catalysts were used to evaluate the metal–support interaction between the active sites (Ni) with the Gd_x_Ce_1-x_O_2-δ_ support. As is shown in Figure 3, all catalysts show a distinct peak in the temperature range of 250–500 °C. NiO reduction typically occurs within the temperature range of 200–500 °C. However, if there is a strong metal–support interaction, the reduction of NiO requires a temperature above 300 °C. In addition, the Gd_x_Ce_1-x_O_2-δ_ support was usually reduced at 300–600 °C. It is difficult to differentiate the reduction peak of NiO with the significant metal–support interaction from that of Gd_x_Ce_1-x_O_2-δ_. However, the metal–support interaction can be evaluated by the number of reduction peaks at temperatures lower than 300 °C. After comparing the H_2_-profiles of the catalysts, it is evident that the reduction peaks of NiO/Gd_0.2_Ce_0.8_O_2-δ_ are mostly located at temperatures above 300 °C, whereas the remaining catalysts exhibit partial reduction peaks at temperatures below 300 °C. In conclusion, NiO/Gd_0.2_Ce_0.8_O_2-δ_ displays the most significant metal–support interaction compared with the other catalysts. Previous studies [28,29] demonstrated that increased oxygen vacancy concentration facilitates enhanced interaction between metal and support. The high metal–support interaction observed in NiO/Gd_0.2_Ce_0.8_O_2-δ_ results from the increased oxygen vacancy concentration, which is achieved through the manipulation of support properties.

### 2.2. Ammonia Decomposition Performance

**Figure 4:**
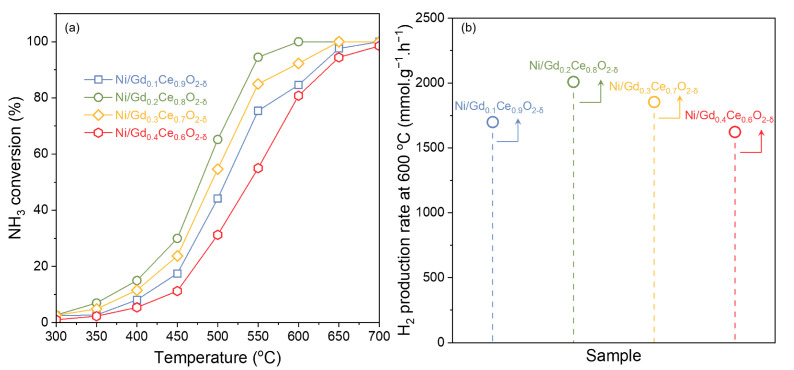
Ammonia decomposition performance over Ni/Gd_x_Ce_1-x_O_2-δ_ catalysts. (**a**) Temperature-resolved ammonia conversion at 300−700 °C, and (**b**) the hydrogen production rate at 600 °C.

The ammonia decomposition performance of Ni/Gd_x_Ce_1-x_O_2-δ_ catalysts was evaluated in the temperature range of 300–700 °C with an interval of 50 °C. As we can see in Figure 4, increasing the decomposition temperature can significantly improve the ammonia conversion for all catalysts. This is because ammonia decomposition is an exothermic reaction, and increasing the reaction temperature enhances its reaction thermodynamics. From a kinetic point of view, an increase in reaction temperature also promotes the rate of ammonia decomposition on the catalyst surface, thus increasing its decomposition efficiency. Comparing the performance of Ni/Gd_x_Ce_1-x_O_2-δ_ with different Gd: Ce ratios, we can observe that the support properties of the catalysts have a significant influence on their catalytic performance for ammonia decomposition. For Ni/Gd_0.4_Ce_0.6_O_2-δ_, the catalyst only started to react at a temperature of 400 °C. Its ammonia decomposition efficiency was only 80.8% at 600 °C. The catalyst’s ammonia decomposition efficiency only approached 100% when the temperature reached as high as 700 °C. For Ni/Gd_0.2_Ce_0.8_O_2-δ_, the decomposition reaction began at temperatures higher than 300 °C, and the reaction efficiency gradually increased to 29.9% at 450 °C, followed by a sharp increase to 100% at 600 °C and GHSV of 30,000 L.kg^−1^.h^−1^ (Figure 4a). At 600 °C, Ni/Gd_0.2_Ce_0.8_O_2-δ_ demonstrated a hydrogen production rate of 2008.9 mmol.g^−1^.h^−1^, outperforming the other catalysts evaluated. The ammonia conversion ranking of the catalysts, from highest to lowest: Ni/Gd_0.2_Ce_0.8_O_2-δ_, Ni/Gd_0.3_Ce_0.7_O_2-δ_, Ni/Gd_0.1_Ce_0.9_O_2-δ_, and Ni/Gd_0.4_Ce_0.6_O_2-δ_. It is worth pointing out that the performance correlates with the oxygen vacancy concentration and Ni dispersion on the surface. 

**Figure 5:**
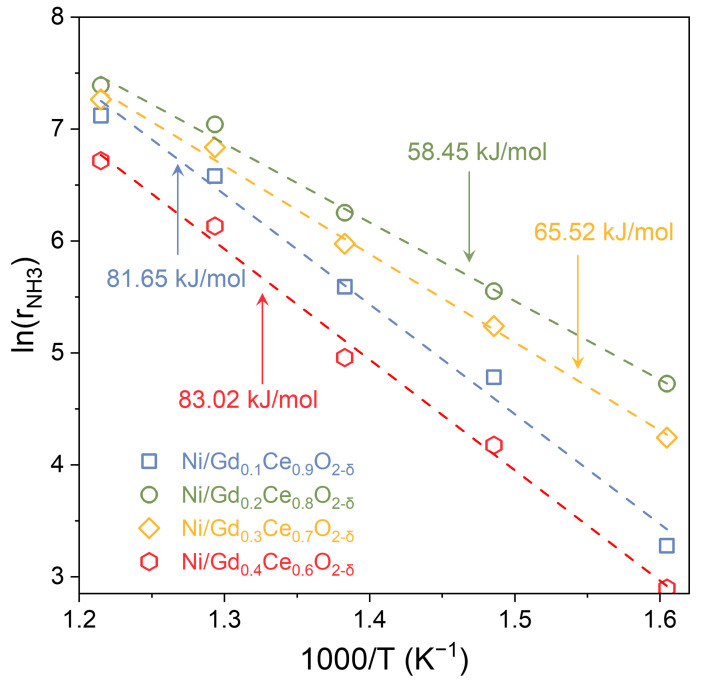
The Arrhenius plots of ammonia decomposition over Ni/Gd_x_Ce_1-x_O_2-δ_.

Arrhenius equation was used to estimate the activation energies:

(2) $$\ln\left(r_{\mathrm{NH}3}\right)=\frac{-E_{a}}{RT}+n$$

(3) $$r_{\mathrm{NH3}}=r_{\mathrm{NH3,catalyst}}-r_{\mathrm{NH3,blank}}$$

where r_NH3_, E_a_, R, and T represent the ammonia decomposition rate, activation energy, molar gas constant, and thermodynamic temperature; $r_{\mathrm{NH3,catalyst}}$ and $r_{\mathrm{NH3,blank}}$ denote the NH_3_ decomposition rate with and without catalysts. The apparent activation energies (E_a_) for the catalysts were evaluated as 81.65 kJ/mol, 58.45 kJ/mol, 65.52 kJ/mol, and 83.02 kJ/mol for Ni/Gd_0.1_Ce_0.9_O_2-δ_, Ni/Gd_0.2_Ce_0.8_O_2-δ_, Ni/Gd_0.3_Ce_0.7_O_2-δ_, and Ni/Gd_0.4_Ce_0.6_O_2-δ_, respectively (Figure 5). We can conclude from the results that Gd_0.2_Ce_0.8_O_2-δ_ support can significantly reduce the apparent energy barrier for ammonia decomposition, resulting in high catalytic performance.

**Figure 6:**
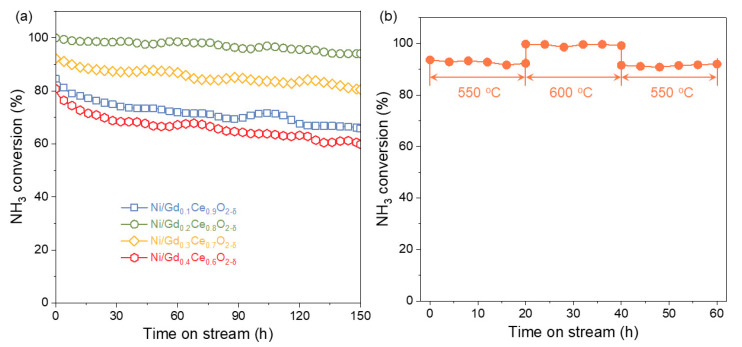
Ammonia conversion as a function of time (**a**) over the course of 150 h and (**b**) at 550–600 °C.

The stability was investigated by conducting the ammonia decomposition reaction over the course of 150 h (Figure 6a). Ni/Gd_0.4_Ce_0.6_O_2-δ_ showed a gradual decrease in ammonia conversion from 80.79% to 59.8%, while Ni/Gd_0.2_Ce_0.8_O_2-δ_ maintained almost constant conversion, indicating its good stability. It can also be found from the results in Figure 6b that the performance of Ni/Gd_0.2_Ce_0.8_O_2-δ_ is reproducible at variable temperatures within the range of 550–600 °C. As we all know, the high stability of the catalysts is derived from the inhibition of aggregation by strong metal–support interaction. These results suggest that adjusting the support properties can boost the concentration of oxygen vacancies, improving the interaction between the metal and support, ultimately resulting in high stability for Ni/Gd_0.2_Ce_0.8_O_2-δ_.

To highlight the novelty of the catalysts in this study, we also compared the performance of Ni/Gd_X_Ce_1-x_O_2-δ_ with those reported in the literature (Table 3). The results show that Ni/Gd_0.2_Ce_0.8_O_2-δ_ exhibits complete ammonia deocmpositon at temperatures as low as 600 °C and high GHSV of 30,000 mL/(g_cat_.h), which is higher than the majority of the catalysts reported in the literature, demonstrating its high potential for practical ammonia decomposition applications.

### 2.3. Mechanical Investigation

#### 2.3.1. DFT Calculation

**Figure 7:**
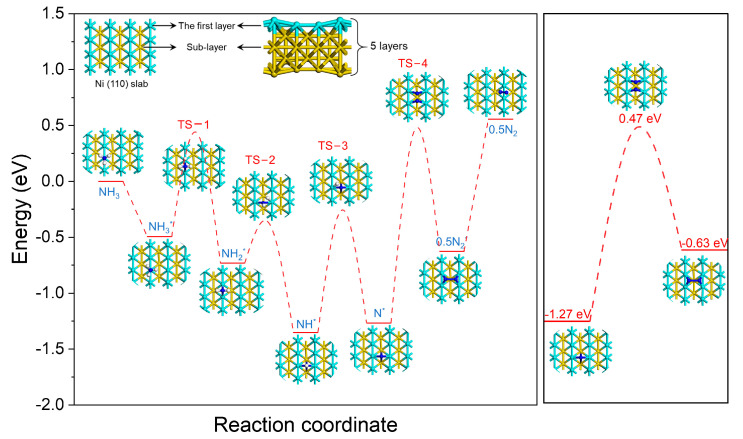
The potential energy profiles of ammonia decomposition over Ni (110) surface.

A DFT calculation was conducted to investigate the reaction route of ammonia decomposition over Ni (110), calculating the potential energy curves for each, thus uncovering the rate-limiting step during this process. The ammonia decomposition reaction was found to consist of two steps, as shown in Figure 7: stepwise dehydrogenation and atom-coupled desorption. A comparison of the energy barriers for these steps revealed that the energy barrier for the associative desorption of N_2_ was estimated to be 1.74 eV, which is much higher than that of the stepwise dehydrogenation processes. Therefore, the rate-limiting step of the ammonia decomposition reaction is the associative desorption of N.

#### 2.3.2. Surface Properties of the Catalysts

Previous studies [32,33] have demonstrated that strong basicity is an important indicator for the enhanced ammonia decomposition activity. Wei et al. [34] further explain the enhanced activity of ammonia decomposition catalysts at strong basic sites. According to Wei et al., the strong basic sites can donate their electrons to the surface of the active sites, benefiting the recombinative desorption of N atoms on the surface. This claim was also supported by many published papers [35,36]. The above analysis has demonstrated that the associative desorption of N is the rate-limiting step. Therefore, evaluating the influence of Gd_x_Ce_1-x_O_2-δ_ support on the abundance of the strong basic sites can be used to explain the enhanced performance of the Ni/Gd_0.2_Ce_0.8_O_2-δ_ at medium temperatures.

**Figure 8:**
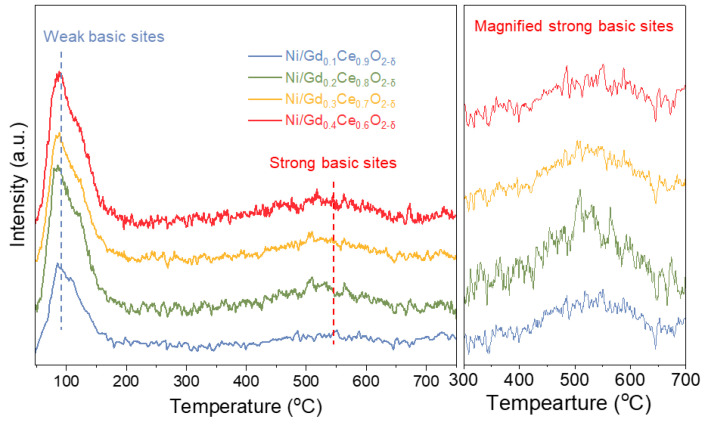
CO_2_-TPD profiles of the Ni/Gd_x_Ce_1-x_O_2-δ_ catalysts.

Figure 8 shows the CO_2_-TPD profiles of the Ni/ Gd_x_Ce_1-x_O_2-δ_ profiles. The spectrum can be classified into two types according to the position of the CO_2_ desorption peaks. The peaks at temperatures <300 °C can be linked to the weak basic sites (Bronstone sites) and the peaks at high temperature (>450 °C) can be ascribed to the contribution of the strong basic sites (Lewis sites). Comparing the CO_2_-TPD profiles of the catalysts, we can find that all catalysts exhibit two CO_2_ desorption peaks at low and high temperatures. In addition, Ni/Gd_0.2_Ce_0.8_O_2-δ_ had the highest area of the peak at a high temperature, followed by Ni/Gd_0.3_Ce_0.7_O_2-δ_, Ni/Gd_0.1_Ce_0.9_O_2-δ_, and Ni/Gd_0.4_Ce_0.6_O_2-δ_. This indicates that Gd_0.2_Ce_0.8_O_2-δ_ support can significantly improve the abundance of the strong basic sites on the surface, benefiting the associative desorption of N on the surface, thus leading to enhanced catalytic performance toward the ammonia decomposition. 

#### 2.3.3. NH_3_ TPSR

**Figure 9:**
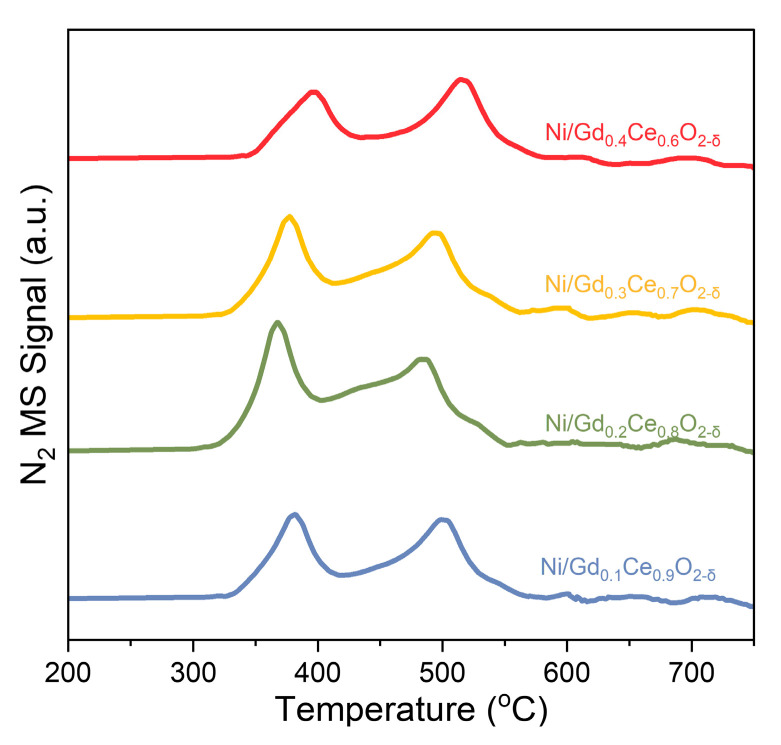
N_2_ formation profiles derived from NH_3_-TPSR measurement over Ni/Gd_x_Ce_1-x_O_2-δ_ catalysts.

NH_3_-TPSR can yield crucial insights into catalyst surface reactions. Our DFT calculations have demonstrated that N recombinative desorption is the rate-limiting step, and the energy barriers can be estimated from N_2_ formation profiles obtained from NH_3_-TPSR (Figure 9). All four catalysts exhibit two N_2_ formation peaks in the temperature range of 300–400 °C and 450–600 °C. A comparison of N_2_ formation peaks reveals that Ni/Gd_0.2_Ce_0.8_O_2-δ_ and Ni/Gd_0.3_Ce_0.7_O_2-δ_ show major peaks at lower temperatures, while Ni/Gd_0.1_Ce_0.9_O_2-δ_ and Ni/Gd_0.4_Ce_0.6_O_2-δ_ have their majority N_2_ formation peaks at higher temperatures. Furthermore, the catalysts can be ranked in order of N_2_ formation temperature as follows: Ni/Gd_0.2_Ce_0.8_O_2-δ_ > Ni/Gd_0.3_Ce_0.7_O_2-δ_ > Ni/Gd_0.1_Ce_0.9_O_2-δ_ > Ni/Gd_0.4_Ce_0.6_O_2-δ_. This ranking indicates that associative desorption of N is easier on Ni/ Ni/Gd_0.2_Ce_0.8_O_2-δ_, which is consistent with its abundant strong basic sites, benefiting the electron conduction to the active sites. Therefore, we can conclude from the results that the Gd_0.2_Ce_0.8_O_2-δ_ support can improve the abundance of the strong basic sites, benefiting the associative desorption of N and, thus, leading to enhanced catalytic performance resulting in ammonia decomposition. The H_2_ and N_2_ signal from the NH_3_-TPSR is shown in Figure S2.

## 3. Materials and Methods

### 3.1. Preparation of the Catalysts

Ni/Gd_x_Ce_1-x_O_2-δ_ were synthesized using a sol-gel method. The support properties were modified by the adjustment of the relative molar concentrations of Gd and Ce. For the synthesis of the Ni/Gd_x_Ce_1-x_O_2-δ_, Ni(NO_3_)_3_·6H_2_O, Ce(NO_3_)_3_·6H_2_O, Gd(NO_3_)_3_·6H_2_O, and citric acid were dissolved in deionized water to form a solution with a metal ion/citric acid molar ratio of 1:1.1. After that, the solution was agitated and heated at 95 °C. The PEG-400 was employed as a gel promoter. The solution was heated at 95 °C overnight to remove all the water in the solution, thus forming the gel. After the evaporation of the water in the solution, the gel was dehydrated and annealed in air at 850 °C for 6 h to derive the final catalysts. The nominal mass ratio of metallic Ni was set as 10 wt%, while the nominal molar ratio of Ce:Gd varied between 9:1 and 6:4.

### 3.2. Characterizations

XRD patterns were obtained using the PANalytical X’Pert-Pro powder X-ray diffractometer with CuKα radiation (0.1541 nm), with a fixed scanning step size of 0.0334° in the 10−80° range, and operated at 40 kV and 40 mA. All catalysts were pretreated in 250 mL/min 10% H_2_/N_2_ for 1 h at 700 °C to reduce NiO before the characterization. The Scanning Electron Microscopy coupled with Energy Dispersive X-ray Spectroscopy (SEM-EDS), Quanta 200FEG with an Oxford EDS system was used for the morphological analysis of the catalysts. An ASAP 2010 instrument (Micromeritics, Norcross, GA, USA) was used to conduct the N_2_ physisorption characterization, which was used to determine the specific surface area of the catalysts. To quantify the actual content, an inductively coupled plasma optical emission spectrometry (ICP-OES) was carried out on an Optima 5300 DV instrument. The metal dispersion of Ni was assessed on an ASAP 2010C chemisorption analyzer (Micromeritics) using the static volumetric H_2_ chemisorption method. It was assumed that each nickel atom would adsorb a single hydrogen atom. 

TPR Win v 1.50 (Quantanchrome Instruments Co., Boynton Beach, FL, USA) was used for H_2_-TPR analysis using 10% H_2_/Ar mixture (25 mL·min^−1^) as the reducing agent. The procedure for conducting the H_2_-TPR reaction was as follows: First, 30 mg of catalysts was placed into the equipment. After that, the catalysts were pretreated at 140 °C under 30 mL/min He for 30 min to remove the possible moisture on the surface. Then, the samples were cooled down to room temperature to initiate the reduction reaction. The temperature was ramped at a rate of 10 °C·min^−1^ from room temperature to 800 °C.

CO_2_-temperature-programmed desorption (TPD) measurement was performed using the same instrument as for the H_2_-TPR. The characterization was conducted to evaluate the distribution of the basic sites on the surface of catalysts. Typically, the catalysts were pre-reduced in 10% H_2_/Ar at 600 °C for 4 h to remove all reducible oxygen, followed by cooling the catalysts to room temperature. After that, the catalysts were exposed to CO_2_ atmosphere at 60 °C for 1 h to adsorb CO_2_. After the termination of the adsorption reaction, the reactor was heated to 100 °C and flushed with He for 1 h to remove the physisorbed CO_2_. Finally, the reactor temperature was heated from 100 °C to 800 °C with a ramp of 10 °C/min. 

The NH_3_-temperature-programmed surface reaction (TPSR) was measured using a homemade fixed-bed reactor. To begin the experiment, the reactor was first evacuated and heated to the desired temperature. Ammonia was then introduced into the reactor at a constant flow rate, and the temperature was gradually increased at 10 K/min. As the temperature increased, the reaction between the ammonia and the catalyst surface occurred, resulting in the production of various products. The effluent gas was continuously monitored by a Pfeiffer Vacuum mass spectrometer.

### 3.3. Computational Details

The Vienna Ab-initio Simulation Package (VASP) was used to conduct density functional theory (DFT) calculations using a five slab-layer Ni(110) and a p(3 × 3) unit cell. The calculations incorporated the Perdew–Burke–Ernzerhof (PBE) functional and the generalized gradient approximation (GGA) to calculate the exchange-correlation energy. The valence electron properties were modeled through the projector augmented-wave (PAW) approach, with an energy cutoff of 400 eV and a Monkhorst-Pack grid of (3 × 3 × 1) used for k-point sampling. To prevent interaction between adjacent slabs, a vacuum spacing of 15 Å in the z-direction was applied. The transition states of ammonia decomposition were determined using the dimmer method, with geometry optimization carried out using the force-based conjugated gradient method. Finally, the adsorption energy (Eads) was calculated as:

(4) $$E_{\mathrm{ads}}=E_{\mathrm{adsorbate}+\mathrm{surface}}-E_{\mathrm{adsorbate}}-E_{\mathrm{surface}}$$

where E_adsorbate_ and E_surface_ denote the total energies of the gaseous species and the clean slab.

### 3.4. Experiment Setup

The ammonia decomposition reaction was conducted in a homemade, lab-scale fixed bed reactor. A quartz tube (300 mm in length, 8 mm in diameter) and a porous distributor were used to support the catalysts. An electric furnace was used to heat the reactor. The temperature of the reactor and the catalyst was controlled and monitored by two thermocouples to achieve the desired operating temperature. The details of the experimental setup used in this study are depicted in Figure S3. Prior to the ammonia decomposition reaction, calcined catalysts (200 mg) were subjected to pretreatment in 10% H_2_/N_2_. The reaction performance was evaluated using pure NH_3_ at 300–650 °C, at the gas hourly space velocity (GHSV) of 30,000 mL/(g_cat_.h). The residual NH_3_ in the effluent was absorbed in a 6 mol/L sulfuric acid solution. A mass flowmeter was used to monitor the flow rate of the outlet gas. 

The NH_3_ conversion was calculated as follows:

(5) $${\mathrm{NH}}_{3,\;\mathrm{conversion}}=\frac{F_{\mathrm{NH}3,\mathrm{converted}}}{F_{\mathrm{NH}3,\mathrm{inlet}}}$$

Since ammonia is converted to N2 and H2 in the outlet gas, and a unit volume of ammonia is converted into two volumes of nitrogen and hydrogen mixture, the volume of ammonia converted is half the flow rate of the gas after the unconverted ammonia has been removed from the reactor outlet. That is, the converted ammonia flow rate was calculated as:

(6) $$F_{\mathrm{NH}3,\mathrm{converted}}=\frac{F_{\mathrm{out}}}{2}$$

Therefore, NH3 conversion can be calculated as follows:

(7) $${\mathrm{NH}}_{3,\;\mathrm{conversion}}=\frac{F_{\mathrm{NH}3,\mathrm{converted}}}{F_{\mathrm{NH}3,\mathrm{inlet}}}=\frac{F_{\mathrm{out}}}{2F_{\mathrm{NH}3,\mathrm{inlet}}}$$

## 4. Conclusions

Tuning the support properties can enhance the activity and stability of Ni/ Gd_x_Ce_1-x_O_2-δ_ for ammonia decomposition to obtain high-purity hydrogen. Ni/Gd_0.2_Ce_0.8_O_2-δ_ shows almost full ammonia decomposition, leading to a high hydrogen production rate (2008.9 mmol.g^−1^.h^−1^) at 600 °C and 30,000 mL/(g_cat_.h). The performance persisted over 150 h long run and at variable temperatures of 550–600 °C. The mechanism investigations have manifested that Gd_0.2_Ce_0.8_O_2-δ_ exhibits high oxygen vacancy concentration, highly dispersed Ni, and abundant strong active sites. These properties of Ni/Gd_0.2_Ce_0.8_O_2-δ_ significantly improve the associative desorption of N and strengthen the metal–support interaction. Therefore, Ni/Gd_0.2_Ce_0.8_O_2-δ_ shows satisfactory activity and stability at 600 °C.

## Figures and Tables

**Table 1:** The crystallite size and lattice constant of the Ni/Gd_x_Ce_1-x_O_2-δ_ catalysts.

	Crystallite Size (nm)	Lattice Constant (Å)
Ni/Gd_0.1_Ce_0.9_O_2-δ_	58	5.15
Ni/Gd_0.2_Ce_0.8_O_2-δ_	60	5.17
Ni/Gd_0.3_Ce_0.7_O_2-δ_	57	5.20
Ni/Gd_0.4_Ce_0.6_O_2-δ_	59	5.23

**Table 2:** The textural properties of the catalysts.

Catalysts	Surface Area ^a^	Actual Ni Loading ^b^	Ni Dispersion ^c^	Ni Particle Size ^c^
	m^2^/g	wt%	%	nm
Ni/Gd_0.1_Ce_0.9_O_2-δ_	121.4	9.85	15.8	8.7
Ni/Gd_0.2_Ce_0.8_O_2-δ_	120.6	9.93	20.7	8.3
Ni/Gd_0.3_Ce_0.7_O_2-δ_	124.3	9.79	17.1	8.1
Ni/Gd_0.4_Ce_0.6_O_2-δ_	119.5	9.85	14.8	8.9

^a^ derived from BET measurement, ^b^ derived from ICP-OES, ^c^ derived from H_2_ chemisorption.

**Table 3:** Comparison of the catalytic activity of Ni/Gd_x_Ce_1-x_O_2-δ_ with those in the literature.

Catalysts	Temperature	GHSV	NH_3_ Conversion	Hydrogen Production Rate	References
	°C	ml/(g_cat_.h)	%	mmol/(g_cat_.h)	
Ni/Al_2_O_3_	700	30,000	79.2	1591.1	[19]
FeNi/Al_2_O_3_	700	30,000	15.7	315.4	[19]
CoNi/Al_2_O_3_	700	30,000	100	2008.9	[19]
CuNi/Al_2_O_3_	700	30,000	34.7	697.1	[19]
Ni/ZrO_2_	650	6000	34.8	139.8	[30]
Ni/TiO_2_	650	6000	83.1	333.9	[30]
Ni/CeO_2_	650	6000	89.7	360.4	[30]
Ni/La_2_O_3_	650	6000	100	401.8	[30]
Ni_10_/CeO_2_	650	30,000	31.0	622.8	[31]
Ni_7.5_Co_2.5_/CeO_2_	650	30,000	31.0	622.8	[31]
Ni_2.5_Co_7.5_/CeO_2_	650	30,000	31.0	622.8	[31]
Co_10_/CeO_2_	650	30,000	31.0	622.8	[31]
Ni/Gd_0.1_Ce_0.9_O_2-δ_	600	30,000	84.6	1699.6	This work
Ni/Gd_0.2_Ce_0.8_O_2-δ_	600	30,000	100	2008.9	This work
Ni/Gd_0.3_Ce_0.7_O_2-δ_	600	30,000	92.3	1854.2	This work
Ni/Gd_0.4_Ce_0.6_O_2-δ_	600	30,000	80.8	1623.2	This work

## Data Availability

The data that support the findings of this study are available from the corresponding author upon reasonable request.

## Supplementary Materials

The following supporting information can be downloaded at: https://www.mdpi.com/article/10.3390/molecules28062750/s1, Figure S1: SEM images of the as-prepared NiO/Ce_x_Gd_1-x_O_2-δ_ catalysts. (a) Ni/Gd_0.1_Ce_0.9_O_2-δ_, (b) Ni/Gd_0.2_Ce_0.8_O_2-δ_, (c) Ni/Gd_0.3_Ce_0.7_O_2-δ_, and (d) Ni/Gd_0.4_Ce_0.6_O_2-δ_, Figure S2: (a) NH_3_ and (b) H_2_ profiles derived from NH_3_-TPSR measurement over Ni/Gd_x_Ce_1-x_O_2-δ_ catalysts, Figure S3: Schematic illustration of the ammonia decomposition setup.
