# Supplementary material for: Enhanced Ammonia Decomposition by Tuning the Support Properties of Ni/GdxCe1-xO2-δ at 600 °C
Source: Molecules. 2023 Mar 18;28(6):2750. doi: 10.3390/molecules28062750 (PMC10059070; doi:10.3390/molecules28062750)
Supplement: Supplementary file 1 [file molecules-28-02750-s001.zip › molecules-2263882-supplementary.pdf]

Supplementary materials

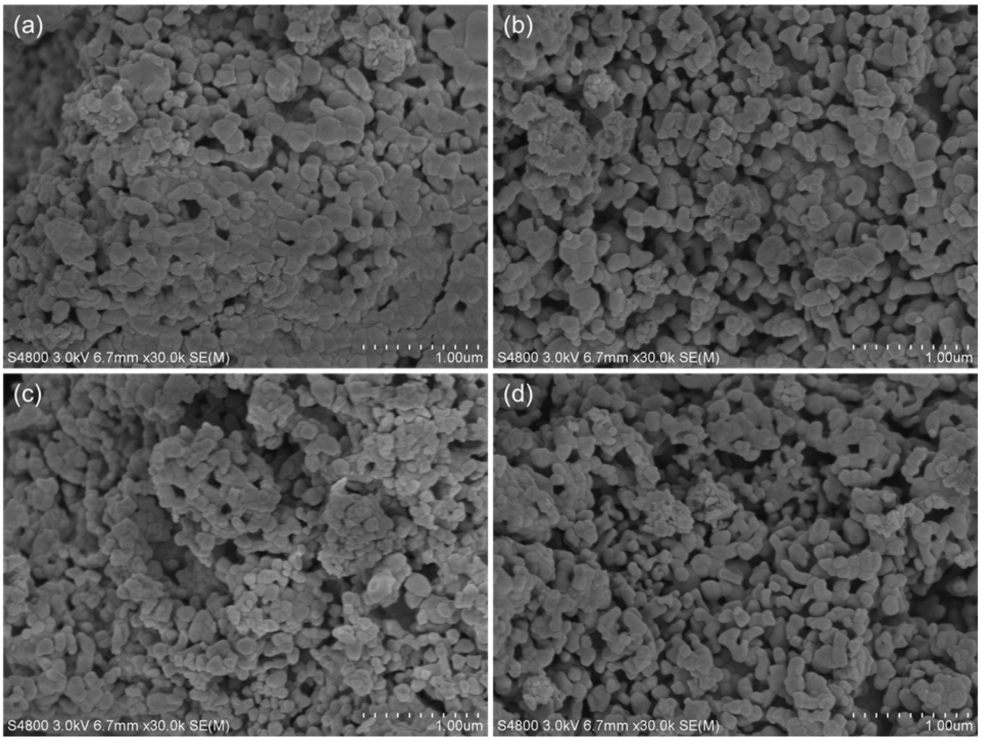

Figure S1. SEM images of the as-prepared $NiO/Ce_xGd_{1-x}O_{2-\delta}$ catalysts. (a) $Ni/Gd_{0.1}Ce_{0.9}O_{2-\delta}$, (b) $Ni/Gd_{0.2}Ce_{0.8}O_{2-\delta}$, (c) $Ni/Gd_{0.3}Ce_{0.7}O_{2-\delta}$, and (d) $Ni/Gd_{0.4}Ce_{0.6}O_{2-\delta}$.

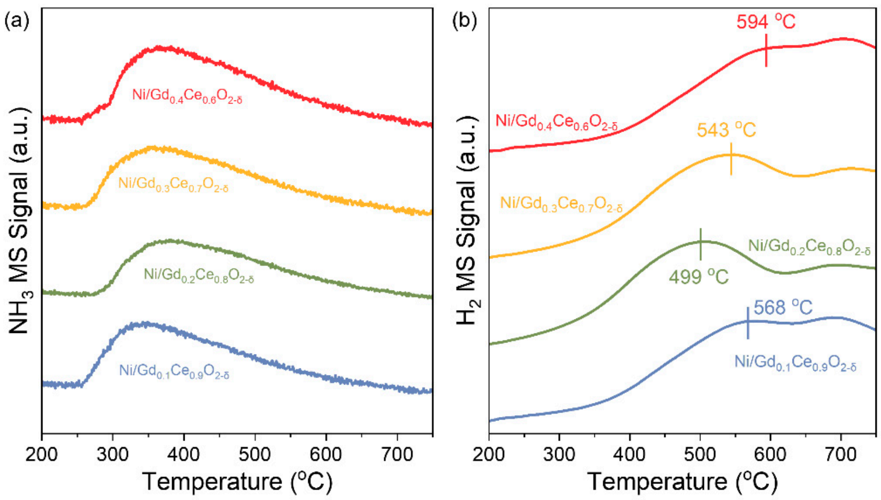

Figure S2. (a) NH_3 and (b) H_2 profiles derived from NH_3-TPSR measurement over $Ni/Gd_xCe_{1-x}O_{2-\delta}$ catalysts.

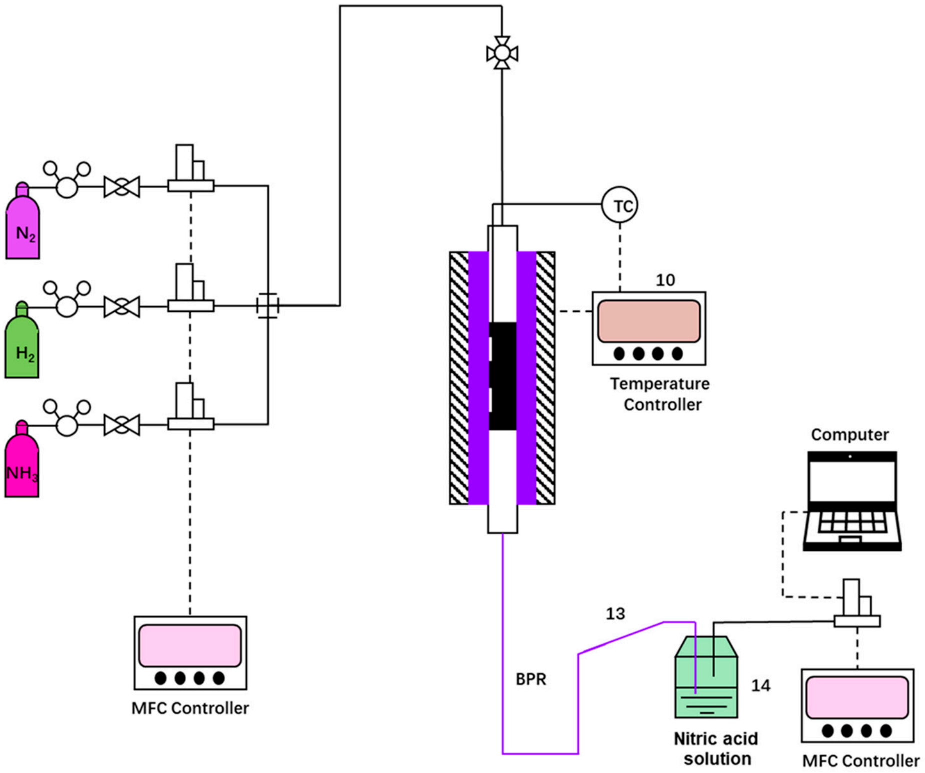

Figure S3. Schematic illustration of the ammonia decomposition setup.
